# Protocol for spike-triggered closed-loop auditory stimulation during sleep in patients with epilepsy

**DOI:** 10.1016/j.xpro.2022.101505

**Published:** 2022-07-31

**Authors:** Hong-Viet V. Ngo, Jan Born, Jens G. Klinzing

**Affiliations:** 1 Department of Psychology, University of Lübeck, 23562 Lübeck, Germany; 2 Center for Brain, Behavior and Metabolism, University of Lübeck, 23562 Lübeck, Germany; 3 Institute of Medical Psychology and Behavioral Neurobiology, University of Tübingen, 72076 Tübingen, Germany; 4 Centre for Integrative Neuroscience, University of Tübingen, 72076 Tübingen, Germany; 5 Bit&Brain Technologies SL (Bitbrain), 50008 Zaragoza, Spain

**Keywords:** Clinical Protocol, Neuroscience, Cognitive Neuroscience, Behavior

## Abstract

Several epilepsies are characterized by interictal spikes in the electroencephalogram occurring preferentially during sleep. We present a closed-loop auditory stimulation protocol with potential for treating sleep epilepsies. We describe the pre-sleep preparations, sleep recordings, the auditory stimulation, in which tones are triggered upon spike detection, and post-sleep procedures. This protocol has been shown to decrease likelihood and amplitude of subsequent spikes in patients with BECTS (Benign epilepsy with centrotemporal spikes) and can be applied to study non-pharmacological treatments of sleep epilepsies.

For complete details on the use and execution of this protocol, please refer to Klinzing et al. (2021).

## Highlights

• Protocol for implementing auditory stimulation in patients with sleep epilepsy
• Tones are triggered upon detection of a spike
• Beneficial effects on patients with BECTS epilepsy
• Potential application as non-pharmacological treatment for sleep epilepsies

## Abstract (editor highlights)

Publisher’s note: Undertaking any experimental protocol requires adherence to local institutional guidelines for laboratory safety and ethics.

## Graphical abstract

**Figure:**
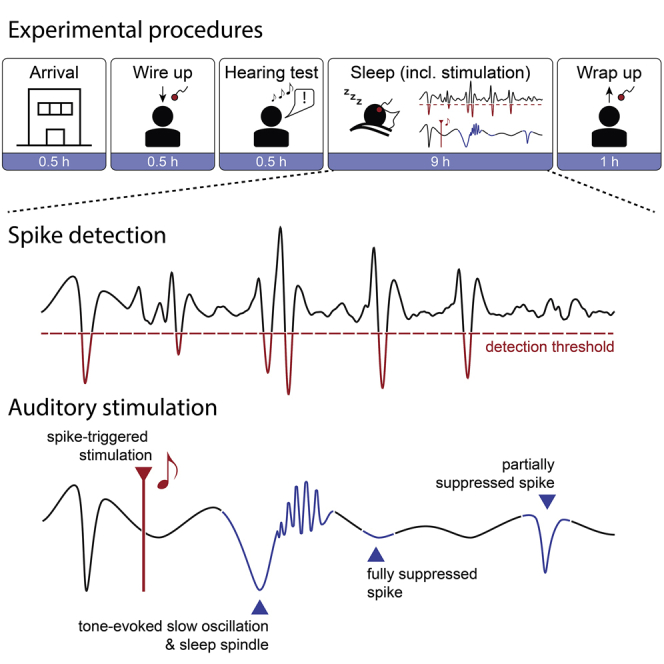

## Before you begin

The following protocol has been implemented by Klinzing et al. (2021) in patients with benign epilepsy with centrotemporal spikes (BECTS). In this study, stimulation led to a reduction in the rate and amplitude of interictal spiking activity. Further analyses suggested this effect may result from acoustically induced activity in the thalamocortical system. There is reason to hypothesize the protocol’s applicability for other sleep epilepsies, such as continuous spike and wave during sleep (CSWS), particularly if an association with sleep spindles or, more generally, an involvement of the thalamocortical system has been demonstrated. While BECTS patients are typically between 6 and 12 years old, expanding the scope of the technique to other conditions may result in the inclusion of vastly different age groups. The method of detecting neuronal events and applying closed-loop stimulation has various conceivable applications outside the realm of epilepsy, which will not be discussed here. We will not describe general questions of obtaining ethics approval, details on informing participants, or participant reimbursement.

***Note:*** The protocol described here only considers the assessment of electrophysiological measures and was developed based on stimulation during a single night. To gauge the clinical relevance of the suppression effect, future studies could incorporate cognitive assessments of before/after or stimulation/sham differences and examine long-term effects of stimulation applied over several consecutive nights.

### Institutional permissions

Studies relying on human patients can be performed only after all necessary institutional permissions have been obtained. Clinical trials should be pre-registered. All procedures must be in accordance with the Declaration of Helsinki.

The study reported in Klinzing et al. (2021) was approved by the local ethics committee of the Medical Faculty of the University Tübingen. Parents gave their written informed consent, patients gave their verbal consent, and both were free to abort the study at any point.

### Planning and participant recruitment

**Timing: weeks to months**

In clinical studies, participant recruitment can be an important bottleneck and often requires the cooperation with one or more clinical partners (hospitals, physicians in private practices etc.).

1. Find clinical partners with regular access to the targeted patient population. In most cases, specialized independent physicians, or medical centers (secondary or tertiary medical care providers) will see the highest number of suitable patients.
  a. Make sure all involved levels of the partner institution (management, head of department, involved doctors, caregivers etc.) are interested in the study, can allocate sufficient time, and have incentives in place to participate over the time of the study. Possible incentives include:
    i. An intrinsic motivation to be involved in a scientific project,
    ii. a co-authorship in the resulting publication,
    iii. the start of a collaboration leading to further research,
    iv. funding opportunities,
    v. and participation in the development of a potential therapeutic intervention.
      ***Note:*** Acknowledging and facilitating these incentives continuously throughout participant recruitment and data collection may promote the swift execution of the research project.
  b. Together with your partners, determine the in-/exclusion criteria for recruiting participants. This is always a tradeoff between the homogeneity of the sample, for which stricter inclusion criteria are more optimal, and greater availability of participants, which is favored by more loose inclusion criteria. Criteria may include:
    i. A diagnosis with BECTS or BECTS-typical centrotemporal spikes,
    ii. the absence of known structural neuronal abnormalities,
    iii. the absence of epilepsy medication,
    iv. a certain age range (e.g., 6–12 years in the case of BECTS) etc.
  c. Together with your partners, assess the likely rate of recruitment and predict realistic time windows for the study. Both may depend substantially on the agreed upon in-/exclusion criteria.
2. Develop strategies and precise procedures for participant recruitment.
  a. Who approaches potential participants first? In Klinzing et al. (2021), this was realized by an attending physician during routine check-ups.
  b. Decide who will be present during the first approach or informed consent discussion.
  c. If appropriate, decide when the scientific investigators take over from the clinicians. What information should be conveyed and how (e.g., the patient’s medical history, incl. details on the epileptic focus and spiking severity).
  d. What medical personnel needs to be present during the trials?
3. Schedule regular re-assessments of the recruitment process with your partners.

### Preparing the sleep laboratory

**Timing: weeks**

4. Choose optimal setup and settings.
  a. The technical setup is explained in detail in the key resources table. Depending on the timing accuracy required for the specific application, simpler setups that include programmable microcontrollers (Arduino, Raspberry Pie etc.) may be sufficient and more flexible. We provide simplified pseudo-code below to allow for platform-independent implementation.
  b. A possible hardware setup comprises a Digitimer D360 amplifier and a CED Power1401 digitizer (Figure 1), for which the following calibration needs to be performed:
    i. To control stimulus presentation, the Power1401 stores the sound stimulus in its internal memory. One can now produce different volumes by scaling the amplitude of this waveform.
    ii. To assess the relationship between each volume setting (i.e., waveform scaling factor) and the resulting absolute sound levels (in dB), measure the sound levels at different settings using a sound level meter (see key resources table) and fit the results with a logarithmic function.
    iii. Invert this function to infer the required scaling factor to a given sound level in dB.

**Figure 1:**
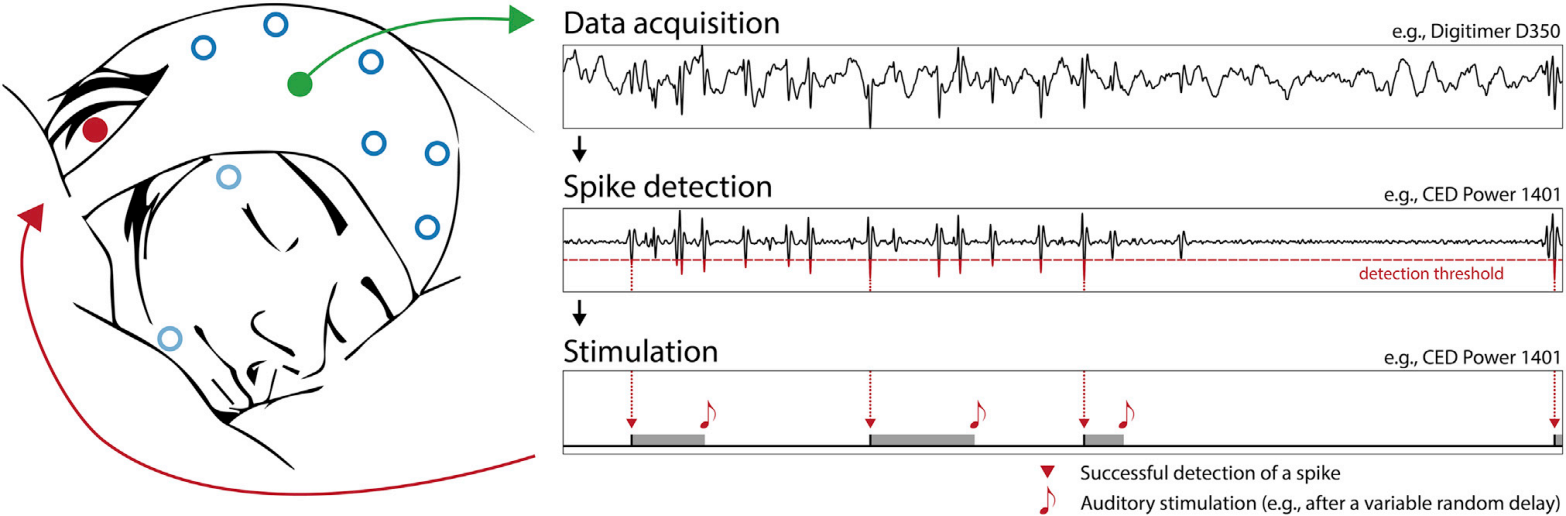
Data acquisition and signal processing The electroencephalographic signal (EEG) used for spike detection is best picked up at a location near the epileptic focus (green). The EEG (see top right trace, “Data acquisition”) is amplified and filtered (e.g., between 4 and 25 Hz), resulting in a signal in which spikes can be detected using a simple threshold procedure (see middle trace “Spike detection”, threshold in red). Once a spike is identified (see bottom illustration, “Stimulation”, red arrows), acoustic stimulation can be triggered with variable delays (gray bars). Note that due to an adjustable pause between stimulations, not every spike triggers a stimulation.
  c. Apart from the technical setup, the sleep laboratory should convey a calm and trustworthy atmosphere. Good mattresses and pillows, colorful bed clothes, cheerful paintings on the walls, rugs, potted plants etc. can help make a cold laboratory cozier and easier to sleep comfortable in. This is particularly important for pediatric patients.
  d. When working with pediatric patients, an additional sleeping option for the parents either in the same or adjacent bedroom is recommended.
  e. As an alternative to the sleep laboratory, mobile setups may be desirable. Using those, stimulation could be performed in a home setting and prolonged auditory stimulation could be assessed while minimizing burden on the patients.
5. Choose optimal montage for neural recordings.
  a. The minimal montage for spike detection requires one electrode in the vicinity of the epileptic focus, a reference electrode at some distance from the affected brain areas (e.g., at the contralateral mastoid), as well as a ground electrode (e.g., at the other mastoid or on the forehead).
  b. The epileptic focus and therefore the optimal location for the spike detection electrode may be known from prior examinations and may be noted in the patient’s medical record.
    ***Note:*** In cases, and when several electrodes are used that cover larger portions of the scalp, the best location may be determined right before the experimental night, based on a recording during quiet wakefulness.
    See troubleshooting 2 for further strategies concerning the detection electrode location.
  c. Detecting sleep onset and assessing sleep stages will require further channels, including electrooculography and electromyography. The exact channel requirements will be determined by the followed sleep scoring criteria, e.g., the AASM manual (Berry et al., 2017) or Rechtschaffen and Kales (1968).
  d. Further channels may be required to assess the topographical distribution of the effects of stimulation on neural activity.
6. Optimal stimulation duration.

To maximize the effect of the stimulation, one may be inclined to perform auditory stimulation throughout the entire night. However, the technique focuses on non-rapid eye movement (NonREM) sleep, which is more dominant in the first half of the night.

***Note:*** The effects of stimulating toward the end of the night, e.g., whether decreased sleep pressure leads to lower arousal thresholds, are a matter of future investigation.

## Key resources table

**Table:** 

REAGENT or RESOURCE	SOURCE	IDENTIFIER
Biological Samples		

14 human subjects (7 girls, 7 boys), mean age ± SD = 9.97 ± 1.52 years (range = 6–11 years)	Klinzing et al., Cell Reports Medicine, 2021	N/A

**Deposited data**		

Analyzed data	Klinzing et al., Cell Reports Medicine, 2021	https://osf.io/pd5x7/

**Software and algorithms**		

Spike2 version 7	Cambridge Electronic Design, Cambridge, UK	https://ced.co.uk/products/spkovin

**Other**		

D360 8-channel patient amplifier	Digitimer Ltd., Hertfordshire, UK	https://www.digitimer.com/product/life-science-research/amplifiers/d360-8-channel-patient-amplifier/
Micro or Power1401 data acquisition interface	Cambridge Electronic Design, Cambridge, UK	https://ced.co.uk/products/mic4in or https://ced.co.uk/products/pow3in
In-ear headphones, e.g., MDR-EX15LP	Sony Europe B.V., Surrey, UK	https://www.sony.de/electronics/in-ohr-kopfhoerer/mdr-ex15lp-15ap
Sound level meter. Appropriate model depends on stimulation setup. Special solutions exist for headphones, e.g., the MiniDSP EARS.	MiniDSP, Hong Kong, China	https://www.minidsp.com/products/acoustic-measurement/ears-headphone-jig

## Step-by-step method details

In case the participants are pediatric patients, as in the case of BECTS, they should be accompanied by at least one parent. There may be situations during the experimental night, in which the child gets impatient, reluctant, or maybe even a bit scared. It is therefore helpful to quickly establish a trusting relationship with child and parent. Allowing the patient to bring their favorite stuffed animal or toy can make the laboratory bed feel less unknown. If the child nevertheless decides not to participate further, this is naturally to be respected. If possible, the parents should sleep in a room right next to the laboratory or on a second bed inside the laboratory.

### Pre-sleep procedures

**Timing: 1.5 h**

Before the sleep recordings begin, follow these steps to prepare the patient and ensure a safe and successful experiment.

1. Participants arrive at the laboratory in the evening, around two hours before their normal bedtime, providing sufficient time for preparations. Take particular care to ensure the child has understood the upcoming procedures and is content with the experiment.
2. In addition to adapting the auditory stimulation volume to the patient’s individual hearing threshold (see step 4 below), the experimenter may choose to screen for previously undiagnosed hearing impairments. To this end, pure-tone audiograms (PTA) may be recorded by assessing the minimal required volume to perceive pure tones of 500, 1000, 2000, and 4000 Hz. Participants with a PTA above 30 dB should be excluded.
3. Apply EEG electrodes and in-ear headphones. Depending on the number of EEG channels, this may take some time and an audio play or active conversation can make this procedure more tolerable for the patient. Make sure the headphones are well secured. Medical tape over the ears and along the cable may be necessary.
4. Assess the individual hearing threshold:
  a. To this end, present a tone to the patient via the headphones. The tone should be the same that will later be used for stimulation (e.g., a burst of pink noise with 50 ms duration and 5 ms falling/rising flank).
  b. In a typical hearing threshold test, the tone is inaudible at first and is stepwise increased in volume until the child signals its perception.
  c. Repeat the procedure to verify the hearing threshold.

***Note:*** For later stimulation, increase the volume by an additional +12 dB to ensure stimulus perception despite increased hearing thresholds during sleep.

**CRITICAL:** Hearing thresholds as measured during wakefulness should not exceed 60 dB SPL, which corresponds to levels during a typical verbal conversation. Higher levels would be indicative of a hearing impairment and could lead to potentially disturbing or even harmful volume settings during sleep.

5. Send patient and, if applicable, parent to bed.

### Sleep recordings and auditory stimulation

**Timing: 3–9 h**

Once the patient reaches stable non-rapid eye movement sleep (NonREM stages N2-N3), when interictal spiking in BECTS typically is at its maximum, present tones to the patient using an automatic or semi-automatic procedure (see script below).

6. Ensure the signal from the currently used detection electrode shows clear spiking with high amplitudes. In case that, even at the optimal location, spikes do not sufficiently stand out from other activity, see troubleshooting 1. In case spikes are more prominent at another location, see troubleshooting 2.
7. Once the subject has reached stable NonREM sleep (i.e., uninterrupted N2 or N3 for a few minutes), start the auditory stimulation at a volume 12 dB above the previously determined hearing threshold. If stimulation causes arousals in the participants, see troubleshooting 3.
8. A possible stimulation setup comprises an analog D360 patient amplifier (Digitimer Ltd.) and a Power1401 data acquisition unit (Figure 1, see also key resources table).
  a. Specifically, the EEG signal at the detection site is recorded and bandpass filtered between 4 and 25 Hz by the D360 amplifier.
  b. This filtered signal is then relayed to the Power1401 unit (Cambridge Electronic Design), which digitizes the signal at 200 Hz and control the auditory stimulation via an in-built sequencer.
9. A key component of the stimulation algorithm is the detection threshold. Stimulation is triggered whenever the recorded signal downward-crosses this amplitude threshold. No fixed value can be provided for the threshold since it is recording-specific and dependent on a variety of physiological, technical, and signal processing factors. These factors include:
  a. The distance of the detection electrode to the epileptic focus,
  b. severity of the discharges,
  c. quality of the recording,
  d. noise levels,
  e. filter settings etc.

***Note:*** Based on our experience, we propose a starting value around -300 μV.

10. The online spike-detection and stimulation algorithm may look as follows:
  a. Phase 1 (Inactive phase): Wait in this phase until the filtered signal reaches a value above the detection threshold (e.g., when returning from a previous spike), then proceed to Phase 2.
  b. Phase 2 (Spike detection and stimulation): Wait while the signal remains above the detection threshold. Once a downward crossing of the detection threshold is detected, deliver an auditory stimulus immediately or after a pre-defined delay. After a stimulus has been presented, the algorithm is briefly paused before it returns to Phase 1. A pause of around 2.5 s is recommended to allow brain responses to unfold and underlying neural refractory processes to expire.
11. Pause detection upon transition into REM sleep, in which spikes tend to disappear in BECTS, or signs of arousals (see below).

***Optional:*** If neural responses to stimulation are intended to be compared with time windows without stimulation, consider adding a period in which spikes are detected and marked in the data, but no tones are presented.

Pseudo-code for a simple stimulation algorithm:

% This algorithm runs in a loop throughout the recording night.

% Parameters:

% s = most recent sample of the EEG signal recorded and filtered

 by the via D360 amplifier

% t = threshold to identify neg. deflections of spikes, default = -300

 μV. Note: threshold requires downward crossing to activate

 stimulation

% d = optional random delay between spike detection and stimulus

 presentation, e.g., 1.5–3.5 s

% r = refractory period following each stimulus presentation

% p = flag coding the current phase of algorithm ([1 2] = phase 1 and 2)

% a = flag representing state of detection with 0 = inactive and 1 =

 active. This flag could be toggled e.g., by an experimenter who is visually assessing the current sleep stage based on the ongoing EEG recording or an online sleep scoring algorithm.

% The algorithm is initialized with a = 1 and p = 1.

if a == 1 {

 % Phase 1: Ensure signal has not yet crossed the downward threshold

 if p == 1 {

 if (s > t) {

   p = 2    % proceed to algorithm phase 2

 }

 }

 % Phase 2: Spike detection and stimulation

 elseif p == 2 {

 if (s < t) {

   wait(d)  % optional random delay

   play sound % auditory stimulation

   wait(r)  % refractory period

   p = 1 % back to algorithm phase 1

 }

 }

}

12. Once activated, monitor the stimulation algorithm for the following cases:
  a. If the stimulation does not induce a visible evoked response in the EEG, e.g., a negative deflection with a trough amplitude of a least -40 μV within 1 s, increase volume by 3 dB SPL.
    ***Note:*** As a safeguard, volume levels should not exceed 80 dB SPL.
  b. If stimulation disturbs the sleeping pattern of the subject, i.e., induces an arousal indicated by an increased EMG amplitude and/or an increase in high frequent activity in the EEG, decrease volume by 3 dB SPL and wait for stable NonREM sleep before stimulation commences again.
  c. If the participant drops out of NonREM sleep, stop the stimulation, and continue only after stable NonREM sleep has re-established.
13. Continue until the planned stimulation duration is reached. Turn off the stimulation equipment. The participants can now continue sleeping without further stimulation. Make sure the child remains able to call for their parent if needed.
14. At a previously agreed upon time, wake up the parent and let them wake the child.

### Post-sleep procedures

**Timing: 0.5–1 h**

In the morning, retrieve all equipment and debrief patient and parent.

15. After the patient is awake, remove the electrodes.
16. Perform a debriefing: Fill out debriefing questionnaire. It is often preferable for either experimenter or parent to go through the questionnaire together with the child in the form of a conversation, rather than in a strict question-answer manner. Make sure the child can say and ask anything it likes. The patients should feel that their contribution is valued by the experimenters and leave with a positive memory of the experiment.

## Expected outcomes

**Figure 2:**
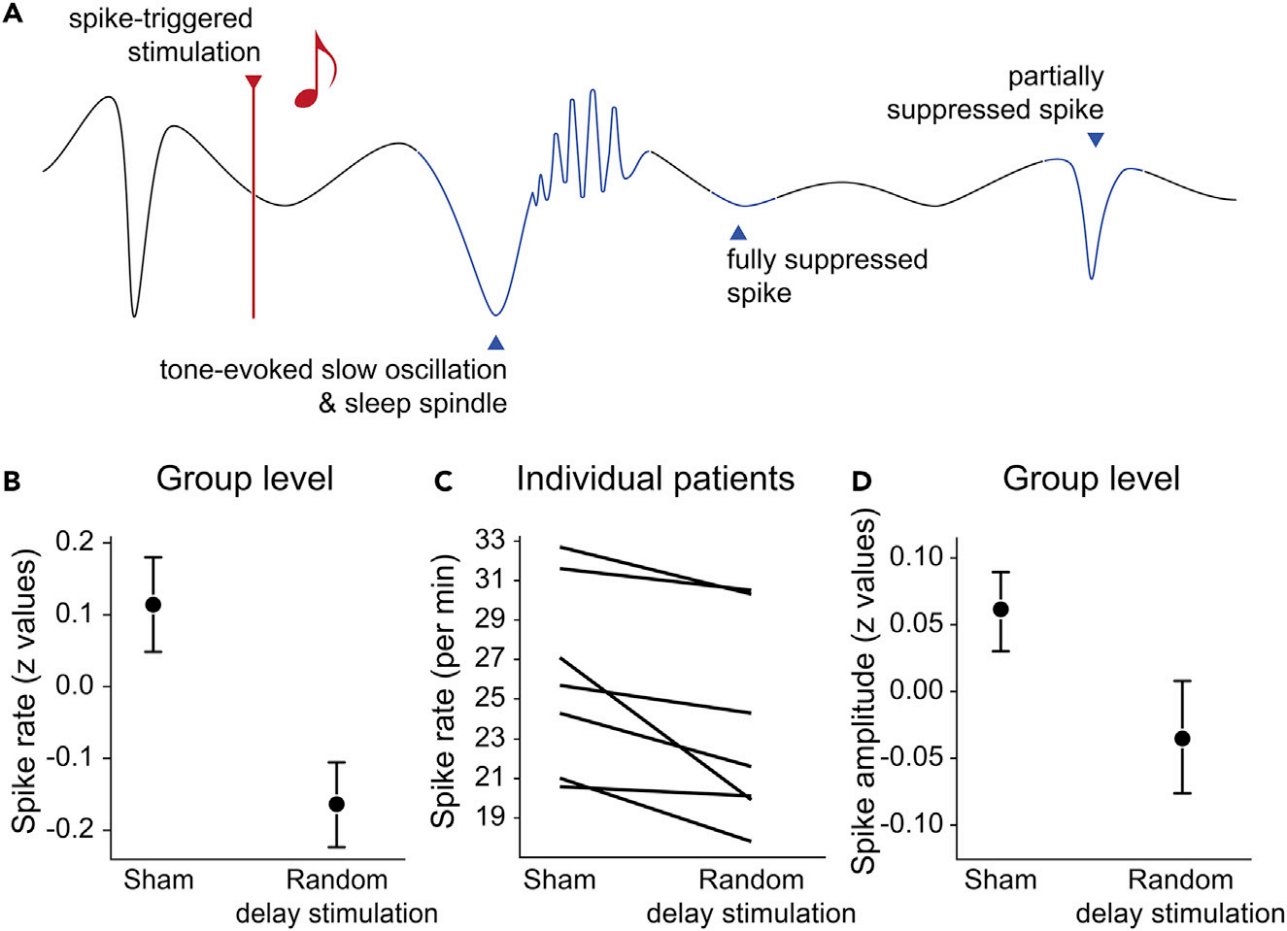
Expected effects of the stimulation (A) Schematic drawing of the effects of auditory stimulation on the recorded brain activity. A detected interictal spike leads to triggering of a stimulation (vertical line with note in red). The presented tone elicits a slow oscillation with an associated sleep spindle nested into the slow oscillation up state. This neuronal event complex leads to the temporary full or partial suppression of further spikes. This model is based on results presented in Klinzing et al. (2021), in which auditory stimulation resulted in a reduced occurrence rate and amplitude of interictal spikes (B–D). (B) Mean ± boot-strapped SEM of normalized spike rates pooled across subjects. (C) Means of non-normalized, non-pooled spike rates for each patient. (D) Stimulation lowered amplitude of spikes still occurring within 1.5 s of the stimulation. While the effects of several stimulation conditions have been examined in this study, here we show results from the condition resulting in the highest reduction in spike occurrence (“Random delay” stimulation condition). Please find further details and results of the statistical analyses in Klinzing et al. (2021).

Results from Klinzing et al. (2021) showed a reduction in the rate of spikes in response to the auditory stimulation in seven children with BECTS (Figure 2). The reduction was most pronounced and statistically significant in a condition in which tones were presented with a variable delay (between 1.5 and 3.5 s) after a detected spike. In addition to a suppression of the occurrence rate of spikes, stimulation also reduced the amplitude of remaining spikes appearing within 1.5 s after the auditory stimulus. In Klinzing et al. (2021), we did not control for further spikes occurring in between the detected spike and presentation of the tone. Therefore, in some cases, the last spike might have occurred closer to the stimulation than the desired delay. Based on our results, we nevertheless concluded that stimulation is likely to be most effective when performed with a delay of 1.5 s or more (i.e., outside a hypothesized thalamocortical refractory period).

## Quantification and statistical analysis

Quantifying the effects of stimulation requires careful analysis of the acquired signals. To optimize detection accuracy, the investigator may choose to use more complex offline spike-detection algorithms than were used online during the recording (as described in the step-by-step method details). In the original publication, Klinzing et al. (2021) assessed stimulation efficacy based on discrete spike events that were identified using such an optimized offline detection algorithm. In brief, an Independent Component Analysis (ICA) decomposition (fastICA) was computed to reject ICA components that did not contain spike-like waveforms. Next, after a back-projection into EEG space, the EEG signal of the detection electrode was high-pass filtered at 5 Hz and the envelope calculated using a Hilbert transform, followed by a smoothing using a moving average with a window length of 50 ms. This allowed the calculation of an individual spike detection threshold based on the mean of the envelope signal +2.5 times its standard deviation. Finally, a spike was registered whenever the enveloped exceeded the threshold for more than 10 and less than 500 m. Putative events following within 50 ms were merged. The values of these parameters (i.e., the offline detection threshold, the minimum and maximum event lengths, and the temporal merging threshold) were chosen empirically based on the data acquired for Klinzing et al. (2021) and should be understood as a broad guidance. They will likely have to be adapted for future studies.

Spike rates and amplitudes were analyzed after pooling across participants and compared between conditions (with the units of observation being the 30-s stimulation blocks). In an additional analysis, data was averaged within each condition and participant, after which conditions were compared across participants. Whereas pooling data across participants increases sensitivity of the analysis by capitalizing on the large number of trials, the second type of analysis can show the reliability of the effect across subjects. An optimal analysis method might involve Bayesian multi-layer statistics that account for both the variance of the rate and amplitude estimates within each subject, as well as variance between subjects. For these types of analysis, it will be beneficial to minimize the number of conditions and maximize the number of trials (i.e., stimulation blocks over which rate and amplitude are calculated). We recommend running simulations to test whether a given protocol is likely to lead to data for which multi-layer statistics are applicable before running the experiment.

Please find further details on data analysis options in Klinzing et al. (2021).

## Limitations

One limitation of the results reported in the original publication (Klinzing et al., 2021) is a moderate overall effect size. Spikes were reduced from an average across patients of 26.452 ± 0.764 events/min in a condition without stimulation to 23.316 ± 0.682 events/min in the Random delay condition. The effect size was likely the result of a rather exploratory paradigm, which was primarily designed to allow detailed analysis of tone-evoked neural response under different conditions and to minimize burden on the participants. Both, stimulation volume and rate could be increased in future experiments to maximize the effect on interictal spiking. An increase in volume will elevate the likelihood of eliciting an evoked neural response. Increasing the stimulation rate (i.e., lowering the inter-stimulus interval) in turn will induce more densely aligned suppression time windows, with less opportunity for interictal spikes to emerge in between. Please note, any modifications of such parameters should be performed with caution and under careful monitoring of arousal levels to prevent disturbing sleep (see “sleep recordings and auditory stimulation”).

The condition with the highest spike suppression (Random delay stimulation) in the original protocol followed in Klinzing et al. (2021) triggered stimulation 1.5–3.5 s after a spike was detected. Spikes occurring within this delay period were not considered. This resulted in a certain fraction of stimulations to occur closer to a preceding spike than the intended delay. Future protocols could incorporate an adaptive delay, for instance by tracking the median inter-spike interval of the last few minutes. Once a spike is detected, stimulation would then be triggered after this adaptive delay or canceled if another spike is detected first. In this latter case, only the delay is updated, and another detection cycle commenced.

## Troubleshooting

### Problem 1

Spikes do not stand out prominently from the background activity during the stimulation night (step 6).

### Potential solution

**Figure 3:**
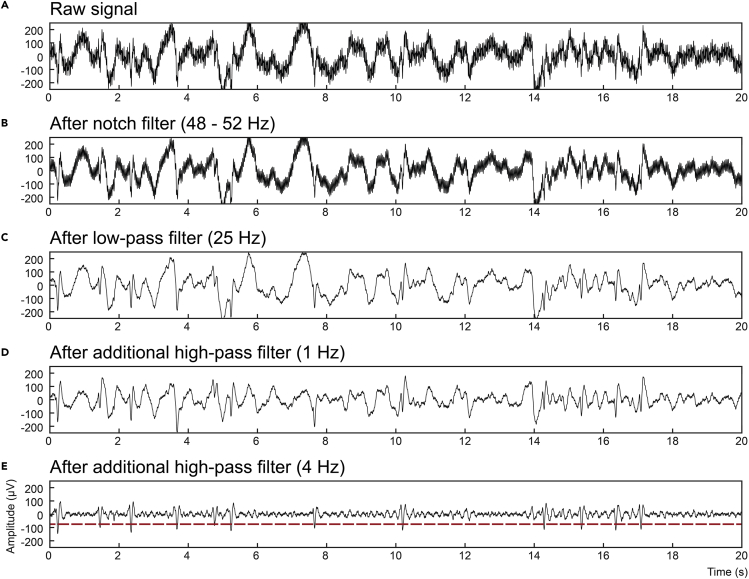
Filtering schemes during spike detection Well-designed filters can aid spike detection during the stimulation night (step 6). (A) Raw signal recorded near to the epileptic focus of a BECTS patient. High-frequency noise at 30 Hz was added to the signal for illustration purposes. The recording is composed of several signal components of both physiological and non-physiological origin. They can hide interictal spikes embedded in the signal, which are supposed to serve as triggers for auditory stimulation. (B) A simple notch filter between 48 and 52 Hz eliminates line noise but does not yet reveal clear spikes. (C) A low-pass filter at 25 Hz greatly dampens the remaining high-frequency noise but low-frequency signal components still hide the target signal. (D) Using an additional high-pass filter at 1 Hz reduces physiological slow oscillations but slow waves at higher frequencies remain the dominant signal component. (E) A stricter high-pass filter at 4 Hz eliminates the slow waves and clearly reveals the interictal spikes. At this point, a simple threshold procedure (red) can be used to detect the target spikes.

Depending on the individual spike morphology and amplitude in relation to the remaining activity, spikes may initially not be easily separable by a threshold procedure. However, to the researcher’s advantage, the spectral content of spikes shows high power in a broad frequency band. Optimal filter boundaries may vary considerable between individuals and with age, scalp location, and epilepsy type. Using recording-specific filter boundaries, e.g., between 4 and 25 Hz, usually allows a clear distinction even from other high-amplitude neural events during sleep such as 0.5–4 Hz slow waves or muscle artifacts at higher frequencies. Please find an example signal snippet and the effect of different filter settings in Figure 3.

### Problem 2

Spikes are visible with highest amplitude at a site other than the detection channel (step 6).

### Potential solution

The epileptic focus may have moved slightly, the information provided be the medical personnel may be inaccurate or differences in the referencing scheme may have led to suboptimal initial positioning of the detection electrode. The recording and stimulation system should thus be set up in a way that the detection electrode can be changed quickly. This is preferably done when the patient is still awake (BECTS spikes often appear at a lower rate already during quiet wakefulness) or in light sleep and before any stimulation has yet been performed.

Depending on the system used, changing the detection electrode may require a change in the recording software or a re-plugging of EEG electrodes. In the latter case, the child should be warned that the experimenter may enter the room at some point during the night.

Please note, in essence problem 1 and 2 reflect inter-individual differences affecting stimulation parameters. This issue can be addressed by inviting participants to an adaptation session or, if possible, by analyzing a pre-existing sleep recording, and assess the most optimal filter settings and detection electrodes before the actual experimental night. Moreover, adaptation sessions allow participants to get acquainted with the environment, experimental procedures (e.g., the wire-up for polysomnography) and experimenters, which helps participants to feel more comfortable and is often reflected in better sleep quality. On the other hand, an additional adaptation session complicates scheduling, increases the required effort, costs, and burden on the patients. Moreover, even if stimulation parameters were extracted from a baseline night or pre-existing recordings, epileptic foci and spike morphology can change over time. Hence, ad-hoc adjustments of these parameters may still be necessary during the experimental session.

### Problem 3

Presentation of auditory stimuli causes arousal or wakefulness (step 7).

### Potential solution

Deriving the stimulation volume based on the above-mentioned approach during wakefulness typically enhances slow wave sleep but may in some cases result in levels exceeding the arousal threshold during sleep. We thus recommend an implementation allowing the option to change stimulation volume during the experiment.

Alternatively, an appropriate stimulation volume can be determined during sleep. To this end, once a patient enters stable sleep for the first time, tones are presented with increasing volume until a K-complex is evoked without signs of arousal (alpha or beta activity).

## Resource availability

### Lead contact

Further information and requests for resources should be directed to and will be fulfilled by the lead contact, Jens G Klinzing (jens.klinzing@uni-tuebingen.de).

### Materials availability

No new reagents or materials are generated using this protocol.

## Data Availability

Algorithms necessary to realize this protocol are provided above as pseudo-code for maximal platform independence. Data generated using the protocol in Klinzing et al. (2021) as well as the statistical analyses based on these data have been uploaded to the Open Science Framework and are publicly accessible: https://osf.io/pd5x7/ (https://doi.org/10.17605/OSF.IO/PD5X7).
